# Antimicrobial Human β-Defensins in the Colon and Their Role in Infectious and Non-Infectious Diseases

**DOI:** 10.3390/pathogens2010177

**Published:** 2013-03-19

**Authors:** Eduardo R. Cobo, Kris Chadee

**Affiliations:** Department of Microbiology, Immunology and Infectious Diseases, Gastrointestinal Research Group, Snyder Institute for Chronic Diseases, Faculty of Medicine, University of Calgary, 3330 Hospital Drive NW, Calgary, Alberta T2N 4N1, Canada; E-Mail: edu.r.cobo@gmail.com

**Keywords:** innate immunity, antimicrobial peptides, β-defensin, colon, intestinal mucosa, parasites, infection

## Abstract

β-defensins are small cationic antimicrobial peptides secreted by diverse cell types including colonic epithelial cells. Human β-defensins form an essential component of the intestinal lumen in innate immunity. The defensive mechanisms of β-defensins include binding to negatively charged microbial membranes that cause cell death and chemoattraction of immune cells. The antimicrobial activity of β-defensin is well reported *in vitro* against several enteric pathogens and in non-infectious processes such as inflammatory bowel diseases, which alters β-defensin production. However, the role of β-defensin *in vivo* in its interaction with other immune components in host defense against bacteria, viruses and parasites with more complex membranes is still not well known. This review focuses on the latest findings regarding the role of β-defensin in relevant human infectious and non-infectious diseases of the colonic mucosa. In addition, we summarize the most significant aspects of β-defensin and its antimicrobial role in a variety of disease processes.

## 1. Introduction

The colonic mucus blanket acts as a substrate for the colonization of indigenous bacteria and in host defense to impede invasion of microbial pathogens. Together with MUC2 mucin secreted by goblet cells, small antimicrobial β-defensin peptides secreted by the colonic epithelium represent the major innate host defenses that serve to maintain a healthy microbiome and ward off potential pathogens [1,2]. Evidence for the importance of β-defensin in the colonic mucosa is seen in inflammatory bowel diseases (IBDs) and infectious colitis, which is characterized by defective expression and/or function of β defensins that lead to altered colonic microflora, microbial invasion, and inflammation [3,4,5,6]. Recently, β-defensins have also been investigated against intestinal parasitic infections by exploring the natural induction of these small peptides during infections and its *in vitro* anti-parasitic activity [7,8,9,10]. However, a role for β-defensins in parasitic infections has not been well explored and will represent a new biologic function for β-defensin either alone or in combination with other innate host mechanisms. This review summarizes the latest findings on the expression of epithelial β-defensin during colonic infectious and non-infectious disorders and the antimicrobial function of β-defensin against important microbes in the gut. This knowledge will help us to understand the complex host-parasite interactions at the site of pathogen colonization and invasion and to explore a role for antimicrobial peptides as therapeutic candidates. 

## 2. Characteristics of β-Defensin Peptides

Defensins are small (4–5 kDa) cationic cysteine-rich peptides of the mammalian innate host defense system [11] that are divided into the subfamilies α, β, and θ based on sequence homology and the connectivity of six-conserved disulfide bonding between cysteine pairs. The primary structure of β-defensins is composed of a mature peptide with 36–47 amino acids residues with a characteristic disulfide Cys^1^–Cys^5^, Cys^2^–Cys^4^, and Cys^3^–Cys^6^ bridge connection [12,13]. In this review we focus on the most recent studies on human β-defensins but it should be noted that other antimicrobial peptides are also component of the innate immune system of the gastrointestinal tract. Human α-defensins, mainly α defensin 5 and 6, are commonly expressed in the small bowel at the base of the crypts of Lieberkuhn, by specialized epithelial cells known as Paneth cells [14]. The other type of antimicrobial peptides relevant in the intestinal tract is cathelicidins. Cathelicidin contains a signal peptide at the *N*-terminus and a more variable cationic region at the *C*-terminal part that possesses antimicrobial activity after cleavage from the holoprotein. The only cathelicidins identified in humans is LL-37/h-CAP18 that is constitutively expressed in various immune cells and inducible in intestinal cells. Other antimicrobial peptides in the innate barrier of the gastrointestinal tract are chemokines CCL14 and CCL15, elafin and secretory leukocyte protease inhibitor that are highly expressed in human epithelial cells [15,16].

## 3. Gene Expression of β-Defensins in the Intestinal Mucosa

In the human genome there are at least 33 β-defensin genes [12,17,18]. Of these, three β defensins, named 1, 2 and 3, have been more intensely investigated in the gastrointestinal tract. Human β-defensin 1 (hBD-1) mRNA is normally expressed at high levels in the colon, followed by stomach and ileum, and at comparatively low levels in the duodenum, jejunum, and tongue [19,20,21]. *In situ* hybridization showed that hBD-1 is present in goblet cells, enterocytes and Paneth cells of the ileum [19]. However, expression of hBD-1 in colonic epithelial cells was unaffected by pro-inflammatory or bacterial molecules [2,22]. Human β-defensin 2 mRNA (hBD-2) was undetected or only minimally expressed in the upper and lower gastrointestinal tract of healthy humans, including gingival keratinocytes, stomach, colon and small intestine [5,19,20,23,24]. Human β-defensin 3 (hBD-3) is also expressed marginally in the small and large intestine [3]. Likewise, human β-defensin 4 (hBD-4) mRNA was expressed at low levels in gastric and small and large intestinal epithelial cells [3,25]. Therefore, it is generally assumed that in the colonic mucosa only hBD-1 is expressed constitutively whereas, hBD-2, hBD-3 and hBD-4 are minimally or not expressed under normal conditions. As discussed below, hBD-2, hBD-3 and hBD-4 are important host defense compounds since they are inducible by inflammatory and infectious stimuli.

## 4. Antimicrobial and Chemotactic Functions of β-Defensin

**Figure 1:**
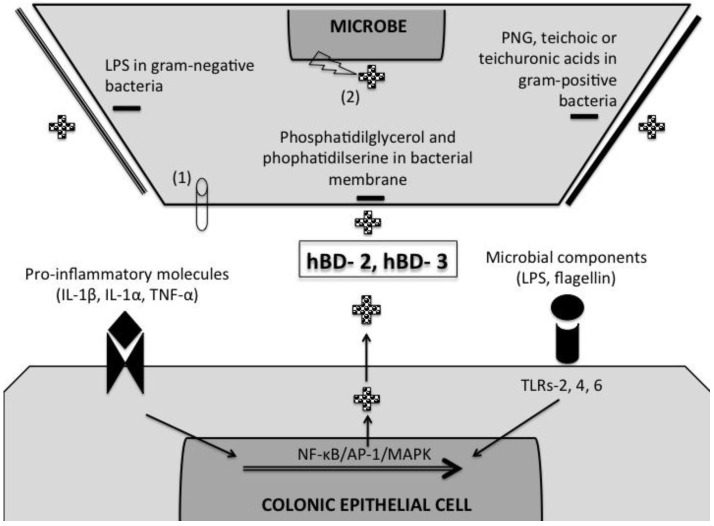
A schematic representation of β-defensin mechanisms of induction and microbial-killing. Pro-inflammatory cytokines (e.g., IL-1β, IL-1α, and TNF-α) and microbial components (e.g., lipopolysaccharide [LPS] and peptidoglycan [PGN]) are capable of inducing β-defensin expression in colonic epithelial cells through specific receptors (e.g., IL-1R, TNF-R and TLRs) and signaling pathways (NF-κB/AP-1/MAPK). The released cationic β-defensins (+) bind negative charged microbial membranes (−). The negative charge of microbial membranes is due to the presence of phosphatidilglycerol and phophatidilserine. In addition, gram-negative bacteria contain LPS and gram-positive bacteria PNG, teichoic or teichuronic acids that charge even more negatively the bacterial membrane. The interaction between β-defensin and microbial membranes may lead to microbial death by permeabilization of the membranes due to pore formation (1), and/or by altering intracellular signaling and inhibiting nucleic acid synthesis (2). The list of pathogens susceptible to β-defensin-killing includes a wide variety of bacteria and protozoa (e.g., *Escherichia coli*, *Pseudomonas aeruginosa*, *Staphylococcus aureus*, *Streptococcus pyogenes*, *Candida albicans*, *Bifidobacterium* spp., *Lactobacillus* spp, *Cryptosporidium parvum*, and *Toxoplasma gondii*).

A key biological activity of β defensins in the gastrointestinal tract is their innate ability to exert microbicidal activity over diverse intestinal pathogens. The antimicrobial mechanisms of β-defensins are initiated by an electrostatic attraction between the cationic antimicrobial peptides and the electronegative charged microbial membranes ([26], Figure 1). The net charge of the microbial membrane is based largely upon its phospholipid stoichiometry and architecture. For instance, bacterial membranes are rich in acidic phospholipids phosphatidilglycerol and phophatidilserine. Moreover, LPS from gram-negative bacteria and teichoic or teichuronic acids of gram-positive bacteria, confers additional negative charge to the bacterial surface (Figure 1). In contrast, host mammalian bilayer membranes are less attracting for antimicrobial peptides because they are rich in phosphatidilethanolamine, phosphatidilcoline and sphingomielin and generally neutral in net charge [26]. This electrostatic affinity may allow antimicrobial peptides to rapidly localize and accumulate at sites of infection, due to preferential affinity for target microorganism surfaces rather than host tissues. Thus, within a certain range, increasing peptide cationicity is associated with increasing antimicrobial potency although excessively strong peptide interactions with phospholipid head groups may decrease antimicrobial activity by preventing translocation of the peptide into the cell interior [27]. 

After the initial electro-attraction, the hydrophobic face of the amphipathic antimicrobial peptides is inserted into the lipid bilayer and the charged arginine side chains bind the polar lipid head groups to form trans-membrane channels. The entry of cationic peptides into the polar target membrane core provokes exaggerated dissymmetry and phospholipid remodeling of microbial membranes [28]. Upon phospholipid translocation or relaxation of the pore, β-defensins are transported from exoplasmic to the inner cytoplasmic membrane facets [26]. Thereafter, the interaction of β-defensins and the microbe surface causes leakage of ions and metabolites, ensuing depolarization, loss of membrane-coupled respiration, impaired peptidoglycan synthesis integrity and ultimately cell death. Microbial death may be preceded by membrane permeabilization due to the formation of channels or a high diffuse accumulation of peptides on the target surface that induce phospholipid displacement changes in membrane fluidity and/or reductions in the barrier properties ([26,29] (Figure 1). Interestingly, antimicrobial peptides can also damage microbes by non-membranolytic mechanisms, including binding to strong negative charge nucleic acids and direct inhibition of nucleic acid synthesis ([30], Figure 1). Thus, it is not clear if membrane permeabilization alone is sufficient to cause cell death or if it is due to disruption of intracellular processes. One aspect that may occur *in vivo* derived largely from *in vitro* studies, is that different antimicrobial peptides (α and β-defensins, cathelicidins) may have multiple and synergistic complementary mechanisms of action to inhibit or kill a wide variety of pathogens in diverse physiologic settings that include growth phase, tissue localization and the presence or absence of other immune mechanisms. For example, within the restricted confines of the phagolysosome, defensins and other antimicrobial peptides are present in relatively high concentrations, where they act harshly and synergistically with one another, along with oxidative killing mechanisms [26,31]. In addition, in the small intestine, secreted α-defensins with antimicrobial activity against commensal and pathogenic bacteria were confined to the crypts and mucus layer, whereas only minute amounts of activity were noted in the luminal content [32]. Curiously, the specific localizations of β-defensins in the colonic mucosa and/or its interactions with the mucus barrier remain unknown.

The antimicrobial activity of β-defensins may determine the fate of several infections of the colon. hBD-2 was shown to be highly effective in killing gram-negative enteric *E. coli* and *Pseudomonas aeruginosa* and yeast *Candida albicans* but it was only bacteriostatic against gram-positive *Staphylococcus aureus* [33]. Similarly, hBD-3 revealed antimicrobial activity against some enteric pathogenic gram-positive *S. aureus* and *S. pyogenes*, as well as gram-negative *P. aeruginosa* and *E. coli* and the yeast *C. albicans* [34]. In the case of hBD-1, it has ubiquitous distribution but poor *in vitro* antimicrobial effect in its oxidative form. However, reduction of disulphide-bridges makes hBD-1 a potent antimicrobial peptide against *C. albicans* and anaerobic gram-positive commensals such as *Bifidobacterium* and *Lactobacillus* species [35]. It is necessary to consider that the *in vivo* killing capacity of β-defensin will depend on the ionic concentration and presence of other innate components in the niche in which pathogen and antimicrobial peptides interacts (e.g., at the base of the crypts). For instance, high concentrations of sodium chloride impair the antimicrobial effect of hBD-2 against *E. coli* [23]. This is due to competitive inhibition by increasing the ionic strength whose solute anion and cation shield the opposing charges of the cationic polypeptides and anionic microbial surfaces and diminish their mutual attraction [36]. On the other hand, the presence of lysozyme and lactoferrin increases hBD-2 killing of *E. coli*, *P. aeruginosa*, *Enterococcus faecalis* and *S. aureus* [23]. Taken together, these studies suggest that the antimicrobial activities of β-defensins are important in protecting the colonic epithelium from virulent pathogens and in regulating the commensal microbiota. 

The final outcome between antimicrobial peptides and microbes also relies on the resistance of microbes to confront and survive antimicrobial peptide-mediated host defenses. Microbes may resist a given antimicrobial peptide by constitutive (passive) or inducible (adaptive) mechanisms of resistance. Among inherent resistance, microbes may constitutively express phospholipid membranes or elaborate capsules/biofilms with reduced electronegativity or composed of an anionic complex of carbohydrate and phosphate that sequester cationic antimicrobial peptides [26]. For example, *Salmonella typhimurium* were showed to evade antimicrobial peptides by increasing aminoarabinose and decreasing anionic charge of lipopolysaccharide. Similarly, some gram-positive bacteria were shown to decrease membrane negative charge by modifying the negatively charged cell wall component, teichoic acid [26,36]. In addition, pathogens may suppress or resist antimicrobial peptide by assuming a dormant metabolic status or preferentially colonizing tissues that have osmotic or ionic strength incompatible with β-defensin activity [26,36]. Other mechanism of resistance includes over expression of proteases and enzymatic modification of surface structures, including O-specific glycosylation that actively protects microbes against antimicrobial peptides [26,36].

Another function of β-defensins is to regulate the adaptive immune responses in the colonic mucosa by chemoattracting immature dendritic cells and memory T cells to mucosal sites against microbial invasion [37]. hBD-2 selectively chemoattract immature dendritic cells and memory T cells expressing the chemokine receptor CCR6, a member of the seven-transmembrane G protein-coupled receptor superfamily [37,38]. Likewise, hBD-2 and hBD-3 bind to the chemokine receptor CCR2 and subsequently induce chemotaxis of monocytes, dendritic cells, and macrophage [38,39]. The recruitment of antigen presenting cells was mediated *via* TLR1 and TLR2 in an NF-κB-dependent manner [40]. At present, it is not known if β-defensins have chemoattraction activity in the healthy colonic mucosa or in colitis. Human β-defensins may have further protective effects on the intestinal epithelium as hBD-2 increased migration but not proliferation of intestinal epithelial cells *via* CCR6 receptors and the expression of MUC2 and 3 mucin [41]. Therefore, with the knowledge of new functions of β-defensins, these formerly named antimicrobial peptides became a more complex host defense peptide that not only kill microbes but also links innate and adaptive immunity.

## 5. Induction of β-Defensins by Pro-Inflammatory and Microbial Stimuli

hBD-2, hBD-3 and hBD-4 are moderately expressed by the colonic epithelium but are induced by specific pro-inflammatory and microbial molecules mediated through multiple signaling pathways. Pro-inflammatory cytokines such as IL-1β, IL-1α and TNF-α, but not IFN-γ, enhanced hBD-2 mRNA expression in Caco-2 and HT-29 human colonic epithelial cells through an NF-κB mediated mechanism [2,42] (Figure 1). However, other signaling pathways of β-defensin induction are present since IL-1β and TNF-α plus dexamethasone induced hBD-2 mRNA expression by a mechanism independent of NF-κB [42]. Microbial components are also capable of inducing β-defensins. Lipopolysaccharide (LPS) found in the outer membrane of gram-negative bacteria and peptidoglycan (PGN) components of gram-positive bacteria and mycobacteria induce β-defensins through PAMP receptors in the colonic epithelium [43] (Figure 1). LPS induced hBD-2 *via* TLR4 and its accessory molecule MD-2 in an NF-κB/AP-1 and Jun kinase-dependent fashion [43]. Peptidoglycan PGN stimulated transcriptional activation of the hBD-2 promoter in a TLR2 and TLR6-dependent manner *via* NF-κB (Figure 1). The induction of hBD-2 by LPS and PGN was highest in colonic cancerogenic SW480 cells, whereas T84 and Caco-2 cells were poorly responsive to LPS and PGN likely due to low expression of TLR4 and TLR2 complexes [43]. Other bacterial components rather than LPS and PGN may be also responsible of inducing β-defensins. Flagellin of probiotic bacteria (lactobacilli from VSL#3 bacterial mixture and *E. coli* strain Nissle 1917) but not enteropathogenic EPEC *E. coli*, up-regulated hBD-2 in colonic Caco-2, T84 and HT29 epithelial cells *via* NF-κB and AP-1 as well as mitogen-activated protein kinase (MAPK) [22,44,45] (Figure 1). Some pathogenic bacteria, including enteroinvasive *S. dublin* or *E. coli*, are also capable of up-regulating the expression and release of hBD-2 in Caco-2 cells mediated by an NF-κB-dependent mechanism [2]. Interestingly, chemical stimuli associated with the cell cycle are also related with antimicrobial peptide production. Butyrate are short-chain fatty acids produced by beneficial colonic probiotic bacteria and sulforaphane is a phytochemical molecule within the isothiocyanate group of organosulfurs highly concentrated in cruciferous vegetables, especially broccoli (*Brassica oleracea*). Both are inhibitors of histone deacetylase and thus, induce apoptosis and inhibit cell proliferation [46]. But, in addition, both butyrate and sulfoinhibitors induced hBD-2 mRNA expression and protein production in HT-29 cells by signaling *via* MAPK/ERK and NF-κB pathways [46]. 

## 6. β-Defensin Response in Infectious and Non-Infectious Inflammation in the Colon

As discussed above, multiple lines of evidence suggests that β-defensins are involved in several colonic infectious and non-infectious diseases and its antimicrobial activity can be harnessed as a potential therapeutic approach. This section summarizes the current knowledge regarding β-defensin role in relevant diseases that affect the intestinal epithelium.

### 6.1. *Cryptosporidium Parvum*

The intracellular protozoan parasite *C. parvum* is the causative agent of cryptosporidiosis, an intestinal disease characterized by acute, watery, and non-bloody diarrhea. Infection with *C. parvum* begins with ingestion of oocysts that excyst in the intestinal lumen releasing sporozoites that infect the epithelium and the development of new oocysts that lead to cell death. β-defensins were showed to have *in vitro* antimicrobial activity against *C. parvum*. Recombinant hBD-1 and hBD-2 were shown to reduce the number of *C. parvum* sporozoites (~50%) after 24h of incubation *in vitro* [10]. Moreover, the presence of cationic peptides (hBD-1, hBD-2, cathelicidin LL-37) individually combined with a monoclonal antibody against *C. parvum* that inhibits sporozoite attachment and invasion, significantly reduced sporozoite infectivity as compared with the antibody alone [47]. Interestingly, *C. parvum* can differentially regulate β-defensin gene expression. Infection with *C. parvum* did not modulate the expression of hBD-3 but markedly down regulated hBD-1 mRNA expression in HT29 colonic cells, which was constitutively expressed in uninfected control cells [10]. In contrast, hBD-2 was undetectable in uninfected HT29 cells but it was over expressed in response to *C. parvum* infection [10]. Likewise, infection with *C. parvum* in human biliary epithelia cells increased hBD-2 mRNA expression through TLR2 and TLR4 *via* MyD88 and NF-κB pathways [7]. Suppression of some β-defensins in *C. parvum* infection was shown in murine models of infection. Murine mBD-1 mRNA and protein was down regulated while mBD-3, a homologue of hBD-2, was constitutively expressed in the murine rectal adenocarcinoma (CMT-93) cell lines after 24h post-infection [10]. Similarly, expression of mBD-1 in neonatal BALB/c mice, constitutively expressed in uninfected control mice, was lowered at the peak of infection, at 4–7 days post infection. This down regulation of mBD-1 mRNA expression was less pronounced in IFN-γ KO animals than in BALB/c wild-type mice, suggesting that IFN-γ may be partly involved in the down regulation of mBD-1 expression [10]. Infection with *C. parvum* similarly induced expression of enteric β-defensins (EBD) mRNA in the colon and distal small intestine of calves [48]. Thus, *C. parvum* may induce hBD-2 and hBD-3 expression but down regulates other β-defensins as an immune escape strategy but, at least *in vitro*, *C. parvum* sporozoites do show certain susceptibility to β-defensins [10]. 

### 6.2. *Toxoplasma Gondii*

Toxoplasmos is is a protozoan parasitic disease caused by *T. gondii* that is usually asymptomatic in healthy people but causes sickness in pregnant women and immune-deficient individuals. Clinical signs include enlarged lymph nodes, muscle pains and other minor symptoms; but in pregnant woman, infection can cause miscarriage or stillborn baby, or malformation in the brain and eye. The life cycle of *T. gondii* begins when oocysts exit the primary host (cat) in stool, sporulate in the environment, and are ingested by intermediate hosts including humans. Sporozoites invade small intestinal epithelial cells and transform into tachyzoites that travel to other parts of the body *via* bloodstream and further develop into tissue cyst bradyzoites in skeletal muscles, brain, myocardium and eyes. Expression of β-defensin in toxoplasmosis is regulated by the clonal genotypes of *T. gondii* (I, II, and III based on its pathogenicity and replication rate). Slow replicating and less pathogenic type II and type III *T. gondii* tachyzoites, but not the fast-replicating and virulent type I strains, increases hΒD-2 mRNA levels in human ileocecal adenocarcinoma cells (HCT-8) and primary cultured human intestinal epithelial cells [9]. Bradyzoites are potent stimulators of hBD-2 in intestinal epithelial cells [9]. On the other hand, hΒD-3 gene expression was down regulated by all three *T. gondii* genotype strains [9]. Thus, virulent *T. gondii*, in contrast to less virulent strains, may down modulate the expression of hBD-2 and hBD-3 in an attempt to establish an infection. However, human β**-**defensins are capable of killing and/or inhibiting *T. gondii*. Supernatant containing hBD-2 after poly (I:C) induction as well as recombinant hBD-2 decreased the number of type I *T. gondii* and its infectivity in HCT-8 cells in a time-dependent manner [9]. These studies indicate that appropriate doses of β**-**defensins may kill or inhibit *T. gondii* infection to overcome its evasive mechanisms.

### 6.3. *Trypanosoma brucei*

African trypanosomiasis is a sleeping sickness caused by *T. brucei* transmitted by insects. The chronic form of trypanosomiasis presents increased endotoxemia due to endotoxins derived from gram-negative bacteria as a consequence of increased intestinal permeability. Intestinal damage due to *T. brucei* infection was reported in experimental infected mice, in which the small intestine showed disruption of villi, cellular infiltration of the mucosa and submucosa and edema of the lamina propria and lymphatics [49]. Procyclic (insect) and bloodstream forms of *T. brucei* showed some *in vitro* susceptibility to antimicrobial peptides. hBD-1 and hBD-2, and α-defensin cryptdin-4 killed both forms of *T. brucei* (between 17% and 33%) although they had lower antimicrobial activity compared with SMAP-29 cathelicidins and its analogs ovispirin and novispirin and protegrin-1 (up to 95%) [8]. Procyclic were less resistant than bloodstream forms to all antimicrobial peptides, presenting immobility within 5 min of treatment and altered structural integrity after 20 min [8]. However, the concentrations of antimicrobial peptides used in this study (5 μmol/L to 100 μmol/L) were much higher than those sufficient to kill various bacteria, fungi and mycobacteria [8]. Higher resistance of bloodstream form of *T. brucei* to antimicrobial peptides is likely due to a higher expression of surface glycoprotein linked *via* glycosylphosphatidylinositol anchors that may block the attachment of the cationic peptides. Thus, antimicrobial peptides show some *in vitro* trypanocidal activity but only at high doses that are not reached in natural infection [8].

### 6.4. *Giardia lamblia*

Giardiasis is caused by *G. lamblia*, a flagellated protozoan parasite restricted to the small intestinal lumen that causes chronic and debilitating diarrhea, malabsorption and growth retardation in infants and young children. At the present, the role of β**-**defensins in giardiasis remains uncertain although it is known α-defensins cryptidins 2 and 3 can kill *G. lamblia* trophozoites *in vitro* [50].

### 6.5. *Candida albicans* and Other Fungal Infections

*C. albicans* is an opportunistic fungus that may affect the gastrointestinal tract, mostly in immune suppressed humans. *C. albicans* resides on epithelial surfaces as part of the physiological microflora but, under certain conditions, it may cause life-threatening infections. One common manifestation of candidiasis is infection of the epithelium in the esophagus before sepsis develops. This esophagitis induced by *C. albicans* increases hBD-2 *via* NF-κB/AP-1 and hBD-3 *via* EGF receptor (EGFR) and its ligand TGF-α**/**metalloproteinase ADAM17 through MAPK/AP-1 signaling cascade [51,52,53]. Recombinant mBD-3 was shown to kill *C. albicans* (MFC, 100 μg/mL) provoking morphological and structural changes in external and internal membranes of the yeast [54]. Moreover, the antifungal activity of β**-**defensin was synergistically improved in combination with the conventional antifungal drugs itraconazole, amphotericin or 5-fluorocytosine [54]. Likewise, reduced but not oxidized hBD-1 was shown to kill several strains of commensal *C. albicans* [35]. In contrast, β**-**defensin did not show an effect with other fungal infections. hBD-2 alone or in combination with lactoferrin or lysozyme did not inhibit germination or invasion of intestinal epithelial cells by *Encephalitozoon intestinalis*, an opportunistic Microsporidia obligate spore-forming unicellular intracellular fungal parasite that causes chronic diarrhea, malabsorption and wasting [55,56].

### 6.6. *Clostridium difficile*

*C. difficile* causes antibiotic-associated diarrhea and pseudo membranous infectious colitis. Exotoxins toxin A and toxin B represent the main pathogenic factors of this disease. Unfortunately, the role of β-defensin against *C. difficile* infections and its specific antimicrobial activity is not well studied. However, a single study showed that while hBD-1 did not protect culture intestinal cells from toxin A and B-induced cell cytotoxicity, human α-defensins reduced cytotoxicity induced by toxin B, but not A [57]. It is possible that the lack of protection afforded by hBD-1 is because this β-defensin needs to be reduced in their disulphide bonds to become active [35].

### 6.7. *Shigella* spp.

*Shigella* spp. (*S. dysenteriae*, *S. flexneri*, *S. boydii* and *S. sonnei*) is a highly contagious gram-negative enteroinvasive bacterium that causes bacillary dysentery. Intestinal infections with *Shigella* spp. can be fatal in untreated infants and children. *Shigella* spp. is unable to invade intestinal epithelial cells through the apical surface, so it translocates through M cells of the follicle-associated epithelium that covers the lymphoid nodules associated with the colonic mucosa. In the subepithelium, *Shigella* spp. causes apoptosis of resident macrophages, allowing bacterial escape into the tissues and spread basolaterally into epithelial cells. It is followed by cell-cell spread and intracellular growth that eventually destroys cohesion of the epithelial barrier and facilitates further invasion of luminal bacteria and propagation of the infection. Interestingly, early *Shigella* spp. infection or watery diarrhea down-regulated hBD-1 transcripts and LL-37 in gut biopsies [58]. Likewise, virulent *S. flexneri* suppressed transcription of hBD-3, which is especially active *in vitro* against *S. flexneri*, in HT-29 cells and human intestinal xenografts [59]. This down-regulation of hBD-3 by *S. flexneri* was also observed in IL-1β–stimulated colonic cells [59]. By blocking antimicrobial factors expression *S. flexneri* seems to progress deeply and massively toward intestinal crypts, at the early time point of infection [59]. Recruitment of dendritic cells to the lamina propria of infected tissues may be similarly impaired since *S. flexneri* down-regulated expression of chemokine CCL20 gene [59]. It has been postulated that the down-regulation of β-defensin depends on *Shigella* plasmid DNA and MxiE transcriptional activator that interfere with the signal pathway(s) for LL-37 and hBD-3 expression [58,59].

### 6.8. Viral Infections

A common cause of colitis by viral infections is the opportunistic infection with double-stranded DNA *Herpesviridae cytomegalovirus* (CMV) in immune-compromised patients, including those with later stage of human immunodeficiency virus (HIV) disease, newborns or after solid organ transplantation or the administration of immunosuppressive medications [60,61]. CMV colitis in patients with AIDS is characterized with symptoms range from low-grade fever, weight loss, anorexia, abdominal pain, and bloody diarrhea to a fulminant colitis [62]. In addition, CMV colitis is rare in patients with mild-moderate ulcerative colitis and Crohn’s disease but highly prevalent in patients with active or steroid-refractory ulcerative colitis due likely to reactivation of latent CMV during periods of intestinal inflammation [63]. The role of β-defensin in viral infections is not completely known but the antiviral activity of β-defensin may involve several mechanisms, including disruption of viral envelopes and viral glycoproteins [64,65]. In the case of CVM, infection of neutrophils with CMV moderately increased LL-37 mRNA levels and this expression of LL-37 transcripts was potentiated in the presence of leukotriene LTB4 [66]. LL-37 demonstrated antiviral effect over CVM mediated by LTB4 since neutralizing LL-37 antibodies partially abrogated the reduction in viral load by LTB4 -mediated neutrophil supernatant [66]. Regarding HIV, hBD-2 and 3 mRNA are induced by HIV-1 in human oral epithelial cells and sero-negative exposed individuals expressed greater mRNA copy numbers of hBD-2 and 3 than controls [67]. Human β-defensin may interact with HIV-1 glycoproteins, such as gp120, and accomplish antiviral functions since hBD-2 and -3 inhibit HIV-1 replication without being cytotoxic to immune-competent cells [65,68]. Other mechanisms of action of hBD-2 and hBD-3 is to inhibit HIV-1 spreading and replication include down-regulation of CXCR4, the co-receptors of HIV-1 [65,68]. On the other hand, β-defensins may serve as adjuvants in anti-HIV vaccines since HIV peptide antigen entrapped with defensins increases HIV antigen specific T-lymphocyte proliferation response at Peyers’ patch and induces a mixed Th1/Th2 type of immune response when administered in mice through intranasal route [69].

### 6.9. Inflammatory Bowel Diseases (IBD)

The term IBD refers to a group of inflammatory diseases of the colon and small intestine. The major types of IBD are Crohn’s disease that affects any part of the gastrointestinal tract but mostly the small intestine and/or the colon and ulcerative colitis that is limited to the colon. Both ulcerative colitis and Crohn’s disease are influenced by multifactorial causes (genetic, environmental and microbial) and may display different degree of inflammation and symptoms. IBD is associated with abnormal production of β-defensins. In ulcerative colitis, there is increased expression of hBD-2, hBD-3 and hBD-4 mRNA in colonic epithelial cells [3,5]. Over expression of hBD-2 and hBD-3 mRNA in ulcerative colitis was correlated with increase expression of the inflammatory cytokines IL-8 and TNF-α mRNA [6]. Interestingly, no increase in β-defensins was observed in colonic epithelial cells from patients with Crohn’s disease [3,5]. Moreover, children with Crohn’s disease showed a lower expression of hBD-2 in the inflamed terminal ileum and ascending colon [6]. In the case of hBD-1 mRNA expression, it was marginally decreased in both Crohn’s disease and ulcerative colitis [5]. The decreased production of β-defensins in Crohn’s disease may respond to individuals carrying mutations in the nucleotide-binding oligomerization domain protein 2 gene (NOD2/CARD15) gene. The NOD2/CARD15 is a cytosolic protein expressed by antigen-presenting cells, Paneth cells and other epithelial cells that is involved in intracellular recognition of microbes by sensing peptidoglycan fragments (e.g., muramyl dipeptide) [70]. Thus, cells over expressing a mutated NOD2 variant associated with Crohn’s disease failed to induce hBD-2 *via* NF-κB [70]. On the other hand, individuals with colonic Crohn’s disease may have diminished β-defensin expression due to a lower hBD-2 gene copy number in the β-defensin locus compared with healthy individuals or patients with ulcerative colitis [71]. Necrotizing enterocolitis (NEC) is an inflammatory disease of the newborn characterized by necrosis of the intestine that affects premature infants. Although the etiologic agent is unknown, extremely low birth weight and the presence of *P. aeruginosa* are considered high risk factors. Moderate cases of NEC in extremely-low-birth-weight infants showed increased expression of hBD-2, whereas severe cases were accompanied by low hBD-2 and TLR4/MD2 expression [4]. This suggests that low expression of hBD-2 may favor the growth of *P. aeruginosa* bacteria that are constitutively susceptible to β-defensins [24]. In summary, colonic cells express β-defensins as a responsive mechanism in inflammatory and immune reactions, mostly by activating TLRs and through the NF-κB pathway. Genetic defects such as Crohn’s disease may weaken β-defensin production, which subsequently causes a dysfunctional control of microbes. Some parasites may have evolved mechanisms to down regulate β-defensins as an evasive mechanism. However, most microbes show some degree of *in vitro* susceptibility to β-defensins suggesting that under an appropriate concentration and physiological setting, antimicrobial peptides may kill and/or inhibit a vast majority of them.

## 7. Concluding Remarks

The antimicrobial effects of β-defensins and their interaction with other pathogens that colonize the intestinal mucosa are not well clearly defined. Despite this, the limited studies done to date have revealed distinct patterns of host induction and potential vulnerable microbial structures. These advances may facilitate the discovery and development of novel anti-infective agents from these ancient host defense molecules capable of interacting with pathogenic membranes. Applications of antimicrobial β-defensin peptides or derived mimetic may bring several therapeutic uses against intestinal infections. Firstly, β-defensin may reconstitute or amplify the antimicrobial efficacies of conventional antibiotics. This is based on the fact that antimicrobial peptides can permeabilize target microbial membranes and could facilitate conventional agents in overcoming access-based resistance mechanisms. Secondly, synthetic β-defensin may reach high concentrations necessary to kill pathogens that exceed those amounts produced naturally. Therefore, the high affinity of β-defensin for microbial membranes may be useful to target essential components involved in adherence, metabolic/energetic pathways, virulence, or intracellular signaling. In addition, the interaction of β-defensin with microbes may induce strategic microbial response pathways that even non-lethal, may prompt organisms to compromise virulence factor or surface feature expression required for adhesion, colonization, or immune avoidance. Finally, specific induction of β-defensin in certain mucosa by the use of stimulators (e.g., butyrate and/or IL-1) may provide necessary levels of antimicrobial peptides *in situ* to fight the infection locally. Thus, pharmacologic agents related with β-defensin may be developed to target strategic microbial structures or functions associated with suppression of pathogen resistance and potentiating conventional antibiotics against drug-resistant pathogens. The colonic mucosa and the related infections is a relative new scenario in which the mechanisms of antimicrobial peptide action, host-induction and microbial resistance hold many questions that need to be addressed.
